# Clinical relevance and implementation into daily practice of pharmacist-prescribed medication for the management of minor ailments

**DOI:** 10.3389/fphar.2023.1256172

**Published:** 2024-01-24

**Authors:** Noelia Amador-Fernández, Irina Botnaru, Samuel Sebastian Allemann, Véronique Kälin, Jérôme Berger

**Affiliations:** ^1^ Center for Primary Care and Public Health, University of Lausanne, Lausanne, Switzerland; ^2^ Center for Research and Innovation in Clinical Pharmaceutical Sciences, Lausanne University Hospital and University of Lausanne, Lausanne, Switzerland; ^3^ School of Pharmaceutical Sciences, University of Geneva, Geneva, Switzerland; ^4^ Institute of Pharmaceutical Sciences of Western Switzerland, University of Geneva, University of Lausanne, Lausanne, Switzerland; ^5^ Graduate School of Health, University of Technology Sydney, Sydney, Australia; ^6^ Pharmaceutical Care Research Group, University of Basel, Basel, Switzerland

**Keywords:** community pharmacies, community pharmacy services, triage, autonomous pharmacist prescribing, implementation science

## Abstract

**Background:** Autonomous pharmacist prescribing was legally introduced in Switzerland in 2019 with the reclassification from prescription medication to pharmacist prescribing of 105 medications for sixteen indications. Its aim was to limit medical consultations and healthcare costs.

**Objectives:** To evaluate the clinical relevance of the pharmacy prescribing medications compared to the over-the-counter medications (OTCs) and to evaluate its implementation into daily practice.

**Methods:** A comparison was undertaken by clinical pharmacists to evaluate chemical and galenical equivalences between pharmacy prescribing medications and OTCs using compendium. ch and pharmavista. ch. Then, a scoping review was carried out in October 2021 to determine clinical relevance according to clinical guidelines’ recommendations. Clinical relevance was completed by determining if pharmacy prescribing medications were part of a homogeneous therapeutic class (no differences in efficacy and safety considered in clinical guidelines, but rather inter-molecular differences) that included an OTC medication. To identify the most clinically relevant pharmacy prescribing medications, first-line treatments were considered. The implementation into daily practice in Swiss community pharmacies was evaluated through an online questionnaire distributed via e-mail from the national pharmacists’ association and LinkedIn^®^. It included 15 questions divided in: pharmacy demographics, experience on pharmacy prescribing, use of prescribing medications and opinion about the them.

**Results:** Of the 105 pharmacy prescribing medications, 20 (19.0%) were first-line treatments without OTC equivalences. Six of them were OTCs reclassified for safety reasons. Ten medications (9.5%) showed a negative clinical relevance (they were not first-line therapeutic options to support pharmacist when managing patients or considered as to be avoided) compared to the OTCs available. For the questionnaire, 283 pharmacists from the German (40.3%), French (37.1%) and Italian-speaking regions (16.9%) answered. In the previous 6 months, 41.7% pharmacies had delivered 10–50 medications and 30.0% between 1 and 10 medications. In situations where patients could be equally treated with a pharmacy prescribing medication or OTC (with an identical OTC, similar OTC or an OTC for the same therapeutic group): 75.6%, 74.9% and 84.8% of pharmacists, respectively, would have chosen OTCs because it required less documentation and it did not require patients’ payment for the service. In addition, pharmacists’ lack of training was also mentioned as barrier for providing the service.

**Conclusion:** Most pharmacist prescribing medications do not present clinical advantages compared to OTCs. In addition, other barriers for implementation were also pharmacists’ training and patient medications costs.

## Introduction

Minor ailments are defined as “common or self-limiting or uncomplicated conditions which may be diagnosed and managed without medical intervention” (Jones et al., 2010). Examples of these conditions are allergic rhinitis or heartburn. In Switzerland, such conditions can be managed in community pharmacy with “over-the-counter (OTC)” products and medications autonomously prescribed by a pharmacist. Similarly, in countries such as United Kingdom or Canada pharmacist are allowed to act as supplementary or independent prescribers for certain health problems including minor ailments (Aly et al., 2018). These services have proven good clinical (Paudyal et al., 2013; Watson et al., 2015; Dineen-Griffin et al., 2020a) and economic outcomes (Rafferty et al., 2017; Dineen-Griffin et al., 2020b; Amador-et al., 2021).

Autonomous prescribing is defined as the act that occurs when “a prescriber undertakes prescribing within their scope of practice without the approval or supervision of another health professional” (Ahpra, 2019; Ogundipe et al., 2023). In Switzerland, autonomous pharmacist prescribing (PP) is allowed in some specific clinical situations, e.g., in order to avoid a direct risk for the patient. Federal laws were revised to broaden PP in order to address the lack of general medical practitioners (GPs), the need to facilitate access to primary care in case of minor ailment (Pharmasuisse, 2021) and to increase patients’ self-care (FGSC, 2022). The Therapeutic Products Act (TPA) was revised in January 2019. Through this revision, a reclassification of medications was introduced stating that pharmacists could dispense, without a medical prescription, medications intended to be delivered under medical prescription. To do so, pharmacists must have direct contact with the patient and they must document the medication dispensed when the medication its indication had been designated by the Federal Council (FOPH, 2019). These medications and indications were defined by a group of experts, consisting of community pharmacists and GPs, and were named as the “list of indications and medicinal products under medical prescription which may be directly supplied by pharmacists” (further called “PP list” in this article).

The PP list has two different medication subcategories: those that were previously under prescription that could now be prescribed by pharmacists (e.g., sildenafil or topical ivermectin) and those that were non-prescription medication and were reclassified for safety reasons as prescription medication that could also be prescribed by pharmacists (e.g., domperidone or doxylamine) (FOPH, 2019). Community pharmacists can dispense medication included in the PP list for sixteen minor ailments (October 2021): seasonal allergic rhinitis, eye diseases, acute diseases of the respiratory system, diseases of the digestive tract, dermatitis, urogenital tract diseases, acute pain, migraine crisis, vitamin and mineral deficiencies, caries prophylaxis, difficulty falling asleep, low blood pressure, travel sickness and vertigo, emergency contraception, opioid overdose and smoking cessation. These are further divided into 43 indications (e.g., rhinitis, bronchospasms or cough for acute diseases of the respiratory system) and 41 therapeutic classes. A medication can have more than one indication (e.g., bilastine for seasonal allergic rhinitis and urticaria) and one indication can be treated by more than one therapeutic class (e.g., seasonal allergic rhinitis can be treated with antihistamines or corticoids).

Regarding the cost that might influence patients when choosing the setting for treating one of the sixteen health problems mentioned, it depends on the provider (Table 1) and on the patient’s co-payment with the Swiss mandatory health insurance:

- Those patients with a lower monthly health insurance bill (around CHF 400, USD 433) are generally people in good health. However, they can pay the maximum yearly co-payment when they are sick (it can go up to CHF 2500, USD 2708) as they need to pay for their medical consultations and medications.
- Even when those patients are paying the maximum amount for the medication and the PP service, their payment is lower compared to the price when consulting a GP. This is a way of switching consultations from GPs to community pharmacists, at least for people in good health, to address the lack of GPs in the health system.

**TABLE 1:** Prices and reimbursement for the consultation and medications of each one of the sixteen minor ailments included in the PP list.

Provider	Pricing			Reimbursement
	Consultation to get the prescription	Medication	Validation of the prescription by the pharmacist	
General practitioner	Fixed price (CHF 60/20 min) (USD 67/20 min)	Fixed price depending on the medication	Fixed prices for validations: prescription (CHF 4.30) (USD 4.80) and medication (CHF 3.60) (USD 3.99)	Yes, depending on the yearly patient’s co-payment
Community pharmacist	Price freely determined by each pharmacy (usually a flat rate of CHF 20–30) (USD 22.30–33.50)		No charge	No

By establishing the PP list, the Swiss government have moved forward to PP. Nevertheless, the clinical relevance of the current list of medications compared to existing OTCs for such activity should be evaluated to determine whether this legal changes support pharmacists with new therapeutic options. In addition, the use of these medications by Swiss community pharmacists, notably compared to OTCs, needs to be explored. This study aims to evaluate both objectives, the clinical relevance of the medications included in PP list compared to OTC medications and to analyze how the use of medications from the PP list have been implemented in daily practices in community pharmacy since the law changed in 2019. This study is of interest beyond Swiss practice, as many countries are aiming to support the management of minor ailments in community pharmacies by developing PP and going beyond the delivery of OTCs.

## Materials and methods

### Objective 1. to evaluate the clinical relevance of the medication included in the PP list compared to OTC medications

A scoping review was carried out by clinical pharmacists in October 2021 to summarize the current evidence of each medication included in the PP list and to determine their clinical relevance, based on guidelines ratio and on comparison with medication already available in OTC. Off-market medications (those removed from the market) were first excluded.

#### Identification of identical or similar medications compared to OTC

A comparison between all medications included in the PP list and those available as OTC in October 2021 was undertaken to evaluate chemical and galenical equivalences. To compare them, usual medication databases in Switzerland were consulted: compendium. ch (HCI Solutions, 2022a) and pharmavista. ch (HCI Solutions, 2022b). The active ingredients were searched by their international non-proprietary names (INN; salts were considered, as these are taken into count in the PP list). Medication from the PP list with identical OTC medications (same active ingredient, dosage and dose form) or similar OTC medications (same active ingredient but different dosage and/or dose form) were considered as having no clinical relevance compared to OTC medications.

#### Evaluation of clinical relevance compared to OTC

The following sources were screened for evidence for each of the indications included in the PP list: National Institute for Health and Care Excellence (NICE), UpToDate, Cochrane Database, Prescrire.org (independent French organization composed by GPs, pharmacists, nurses and dentists), *Revue Medicale Suisse* (independent Swiss organization that is a reference in medical information) and Swiss Medical Society. The search was carried out using each of the indications included in the PP list as keywords. All identified guidelines in English and French were screened. Information about first-line treatments, recommended medication and medication related risks were extracted.

The current medication classification (e.g., prescription or OTC) is made by Swissmedic (Swiss agency for therapeutic products) based on benefit/risk ratio. Therefore, we further evaluated clinical relevance (positive for first-line treatments without an OTC equivalent or in the same therapeutic group or negative for non-first-line treatments or medications to be avoided) based on the utility as new therapeutic options for pharmacists compared to the OTCs already available. Clinical relevance was completed by determining if medications from the PP list were part of a homogeneous therapeutic class, and whether this class included an OTC medication. A therapeutic class was considered homogeneous in case of no differences in efficacy and safety considered in clinical guidelines, but rather inter-molecular differences (e.g., pharmacokinetics). A homogeneous therapeutic class that included an OTC medication determined a lack of clinical relevance for all medications from the PP list.

Finally, to identify the most clinically relevant medications from the PP list, the active ingredients were evaluated to determine if they were considered first-line treatment for the health problems for which they had the indication. When necessary, because of diverging clinical recommendations, consensus was reached by two different community pharmacists from the Centre for Primary Care and Public Health, University of Lausanne, (Switzerland) and a third one in case of disagreement. Not being first-line treatment was also considered a criterion for determining lack of clinical relevance.

### Objective 2. to evaluate the implementation of the PP list for patient’s daily care in community pharmacy

A cross-sectional electronic survey was developed and distributed to community pharmacists for 1 month between 16 September 2021 and 17 October 2021. It was distributed via e-mail from pharmaSuisse (national pharmacists’ association) that counts with 83.3% of all pharmacies in Switzerland as members (Pharmasuisse, 2021) and through LinkedIn^®^. The study did not fulfill the criteria of the Federal Act on Research involving Human Beings (Fedlex, 2022) by the Ethics Committee of Vaud and did therefore not need a formal approval by an Ethics committee given that data was collected anonymously and did not require personal health-related information.

The survey was developed by academic community pharmacists, experts in the field. It consisted of 15 categorical questions that were divided in four different parts: community pharmacy demographics; experience on autonomous prescribing activity; implementation of the medications from the PP list to manage patient’s health problems, and opinion about the current list. The community pharmacy demographics included questions to determine the location and type of community pharmacy (3 questions). The questions on the experience of prescribing were related to the sources and tools to guide and document the service (8 questions). The implementation of the PP list was evaluated through clinical situations that could be managed in the pharmacy by using medication from the PP list or as OTC with similar clinical relevance (2 questions) (Paudyal et al., 2013). Personal opinion on the importance of the PP list and possible additions to the list were asked (2 questions).

The survey was completed in the REDcap^®^ (Research Electronic Data capture) software (version 10.3.3) (Vanderbilt, 2023) which is a web based interface with a secure data collection that meets the HIPAA (Health Insurance Portability and Accountability Act) compliance standards (CDC, 2022). The survey was translated into the three of the official languages in Switzerland by native pharmacists in each of the languages working in Swiss community pharmacy (to improve contextualization for the different Swiss territories): German, French and Italian (Supplementary Appendix S1). Prior to its distribution, the French version of the survey was piloted by seven community pharmacists. Participation in the survey was voluntary and responses were anonymous. In case of several working places, the respondent pharmacist had to take into consideration the community pharmacy where his/her occupational rate was highest at the moment of completion.

### Data analysis

The questionnaires completed on the REDCap^®^ were exported and analyzed using Microsoft Excel^®^ v2016. Descriptive analyses were carried out, data was presented as relative (%) and absolute (n) frequency for categorical variables.

## Results

### Objective 1. to evaluate the clinical relevance of the medications included in the PP list compared to OTC medications

**FIGURE 1:**
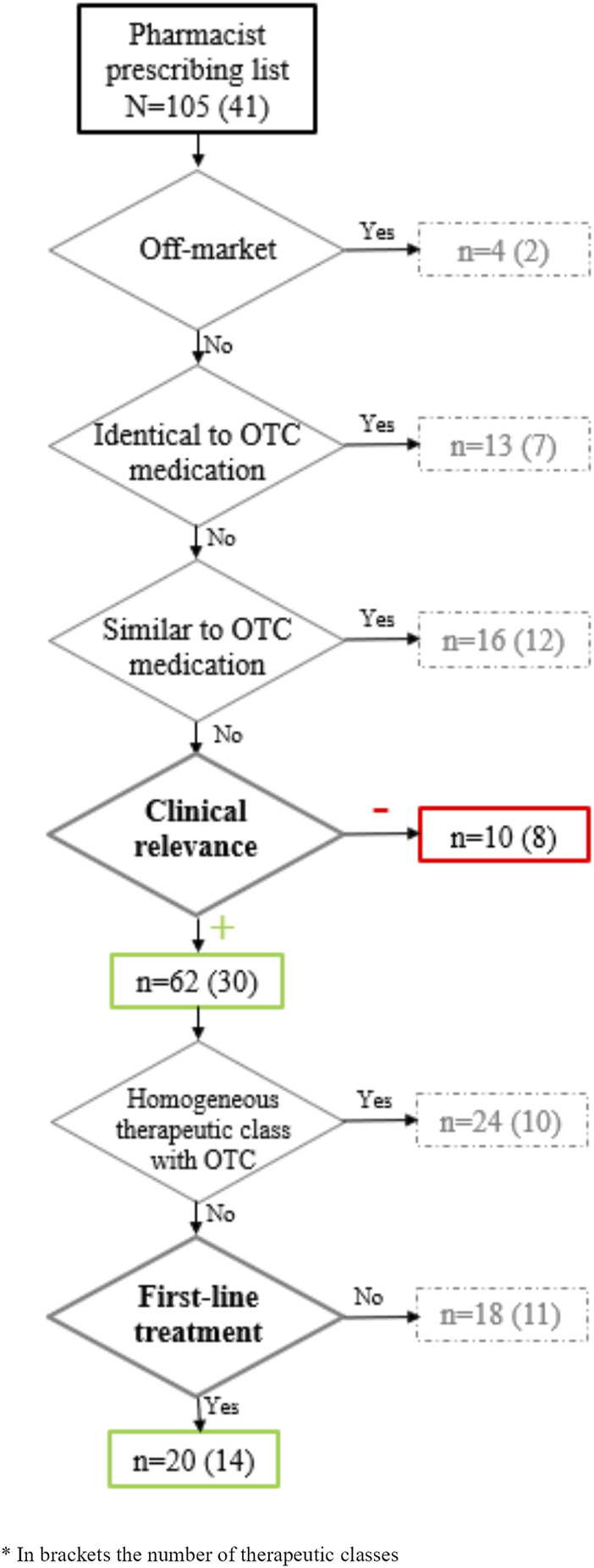
Clinical relevance of the active ingredients included in the pharmacist prescribing list.

As shown in Figure 1, from the 105 medications included in the PP list, 4 medications were excluded (flumetasone 0.2 mg/g ointment, prednisolone 2.5 mg/g ointment, desonide 1 mg/g cream and loratadine 10 mg 28/42tabs), as these were no longer marketed. Some of the medications (n = 13, 12.4%) were considered identical to OTC medications and other medications (n = 16, 15.2%) had similar OTC medications. Ten medications (9.5%) from 8 different therapeutic classes were considered to have a negative clinical relevance. Out of the total medications studied, 62 (59.0%) from 30 therapeutic classes had positive clinical relevance. Among them, 24 medications (22.9%) were considered as part of a therapeutic class that already contain at least one OTC medication. Some of these were: proton pump inhibitors, non-steroidal anti-inflammatory drugs, anti-histaminic, corticoids, antifungals (Supplementary Appendix S2).

Finally, 20 active ingredients (19.0%) and 14 therapeutic classes for 14 indications were determined to be first-line treatments that were clinically relevant, e.g., that provided additional benefits to patients compared to those available in OTC (Figure 1).

Detailed description about all medications analyzed are included in Supplementary Appendix S2.

The 20 medications without an OTC equivalent (e.g., identical, similar or part of a homogeneous therapeutic class including an OTC) found to have a positive clinical relevance that were first choice drug are included in Table 2. These represent 14 indications, as several medications are part of a homogenous therapeutic class that does not include an OTC: 3 topical medications against acne, 3 topical medications against uninfected dermatitis and eczema and 2 medications against migraine. Among these 20 medications, 6 were non-prescription medications that were reclassified for safety reasons as prescription medication that could also be prescribed by pharmacists.

**TABLE 2:** Medications included in the pharmacist prescribing list with no OTC equivalent and a positive clinical relevance.

Medication (INN)	Indication (according to pharmacist prescribing list)	Therapeutic class
Adapalene (topical)	Acne	Antiacne preparation for topical use
Isotretinoin (topical)		
Tretinoin (topical)		
Ivermectin (topical)	Acne rosacea	Other dermatological preparation
Hexamidine diisétionate (topical)^a^	Bacterial conjunctivitis	Antiseptic and disinfectant
Salbutamol (inhalation)	Bronchospasms	Short-acting beta-agonists (SABA)
Terbutaline (inhalation)		
Doxylamine^a^	Difficulty falling asleep	Antihistamine
Levonorgestrel^a^	Emergency contraception	Hormonal contraceptive for systemic use
Naloxone^a^	Emergency treatment of an opioid overdose	Peripheral opioid receptor antagonist
Sildenafil	Erectile dysfunction	Urological
Mebeverine hydrochloride	Functional disorders of the gastrointestinal tract	Drug for functional gastrointestinal disorders
Lidocaine + Prilocaine (topical)	Local anesthesia	Anesthetic local
Naratriptan	Migraine	Antimigraine preparation
Sumatriptan		
Permethrin (topical)	Parasitosis scabies	Ectorapasiticides, incl. scabicides, insecticides and repellents
Cinnarizine^a^	Travel sickness and dizziness	Antivertigo
Clobetasone 17-buthyrate (topical)	Uninfected dermatitis and eczema	Corticoid
Hydrocortisone 17-buthyrate (topical)		
Triamcinolone acetonide + Salicylic acid (topical)^a^		

^a^ Non-prescription medication reclassified for safety reasons as prescription medication that could also be prescribed by pharmacists.

### Objective 2. to evaluate the implementation of the medications included in the PP list into patient’s daily care in community pharmacy

A total of 283 pharmacists completed the survey, out of 5,769 pharmacists who worked in a community pharmacy in Switzerland (4.9% pharmacists in the whole country). Most respondents (40.3%, n = 114) were from the German part of Switzerland, 37.1% (n = 105) from the French speaking regions and 16.9% (n = 48) from the Italian speaking part (missing data for 16 respondents). The type of pharmacies were independent community pharmacies for 42.7% (n = 121) of the pharmacists, 36.4% (n = 103) were part of a group, 19.8% (n = 56) were chain pharmacies and 1.1% (n = 3) were under franchise.

Sources used to get information about the medications available in the PP list are presented in Table 3, with most pharmacists obtaining information from their pharmacy software (55.7%, n = 156). Regarding the support for implementation of medications included in the PP list, over a third of the pharmacists (38.5%, n = 109) answered that they would need additional help to integrate the PP list in their practice. Among these pharmacists, 78 specified the kind of help needed: algorithms (46.1%, n = 36), additional education (21.8%, n = 17), additional documentation (16.7%, n = 13) or a form included in the pharmacy IT system (15.4%, n = 12).

**TABLE 3:** Sources of information and support for implementation in daily practice of medications included in the pharmacist prescribing list.

Source of information	Pharmacist; n (%) (N = 280)
Pharmacy IT system	156 (55.7)
Data sheets from Swiss Community Pharmacy Association	43 (15.4)
Articles provided by a training organization	36 (12.9)
Federal Office of Public Health (FOPH) website	25 (8.9)
Other^a^	12 (4.3)
None	7 (2.5)
Don’t know/Don’t want to answer	1 (0.3)

^a^ Other sources reported: the lecture of internal documents (n = 2), by making documents available (n = 2) netCare (n = 1), other studies (n = 1).

**FIGURE 2:**
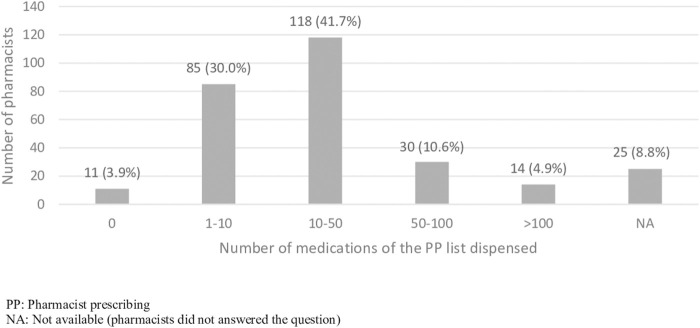
Pharmacies dispensing medications of the PP list 6 months before the survey.

Figure 2 shows the medications dispensed in the pharmacies in the 6 months before the survey, with most pharmacists dispensing between 1 and 10 medications of the PP list (30.0%, n = 85) and between 10 and 50 medications (41.7%, n = 118). Most of the pharmacists who answered (89.8%, n = 254) prescribed these medications themselves.

Most pharmacies that prescribed a medication used an IT platform to document the service (71.1%, n = 180), some documented the process on paper (23.3%, n = 59) and a minority of pharmacies did not record the service (2.8%, n = 7) or used a different method (2.4%, n = 6).

The third area of assessment included in the survey was related to implemented strategies to recommend the medications included in the PP list. Most respondents (66.8%, n = 189) reported that no strategy related to the PP list was implemented in their pharmacy or did not want to answer (7.5%, n = 21). Out of those 73 who confirmed an implemented strategy (52.0%, n = 38 at a pharmacy level and 48.0%, n = 35 at a chain or group level) stated that the service was marketed in their pharmacy (42.5%, n = 31), communication techniques with patients were used such as websites or magazines (31.5%, n = 23), goals were set in terms of numbers of patients (31.5%, n = 23) or communication techniques with other health professionals were used (13.7%, n = 10) (multiple choice was available).

The last part of the survey concerning personal opinion of the pharmacists included their perception of the most important health problems included in the list for their practice (multiple choice). The following health problems were cited in descending order: emergency contraception (72.5%, n = 203), seasonal allergic rhinitis (69.6%, n = 195), eye disorders (62.9%, n = 176), dermatoses (56.8%, n = 159), urogenital diseases (43.9%, n = 123), acute diseases of the respiratory system (38.6%, n = 108) and diseases related to the digestive system (38.6%, n = 108).

In similar clinical situations, when the pharmacist could choose to prescribe identical medications either in PP list or in OTC (e.g., cetirizine or omeprazole), the majority would choose a medication in OTC (75.6%, n = 211). In the occasions where there were similar OTC medications or medication with the same indication as OTC presentation, pharmacists responded likewise: in case of acute pain (74.9%, n = 209) chose a similar OTC medication; or in case of functional disorder of the gastrointestinal tract (84.8%, n = 235) chose an OTC medication with a same indication. The reasons for this choice are included in Table 4.

**TABLE 4:** Reasons for prescribing a medication included in the pharmacist prescribing (PP) list or in OTC in case of similar clinical situations.

Reason to prescribe	In case of identical OTC medication (e.g., cetirizine 10 mg tabs)	In case of similar OTC medication (e.g., acute pain: acetaminophen 1 g VS 500 mg)	In case of OTC medication in the same therapeutic class (e.g., disorder of GIT: mebeverine VS Iberogast^®^)
	OTC: N = 211 (75.6%)	OTC: N = 209(74.9%)	OTC: N = 235 (84.8%)
	PP list: N = 68 (24.4%)	PP list: N = 70 (25.1%)	PP list: N = 42 (15.2%)
It allows to deliver an equally effective medication			
OTC medication	124 (58.8%)	115 (55.0%)	121 (51.9%)
PP list	42 (61.8%)	50 (71.4%)	27 (64.3%)
It is easier			
OTC medication	119 (56.4%)	99 (47.4%)	106 (45.5%)
PP list	45 (66.2%)	35 (50.0%)	24 (57.1%)
It does not require the payment of the service			
OTC medication	114 (54.0%)	93 (44.5%)	94 (40.3%)
PP list	23 (33.8%)	26 (37.1%)	19 (45.2%)
It is faster			
OTC medication	105 (49.8%)	90 (43.1%)	98 (42.1%)
PP list	12 (17.6%)	5 (7.1%)	6 (14.3%)
Other			
OTC medication	12 (5.7%)	35 (16.7%)	38 (16.3%)
PP list	4 (5.9%)	2 (2.9%)	2 (4.8%)

Multiple answers allowed.

*GIT: gastrointestinal tract.

**FIGURE 3:**
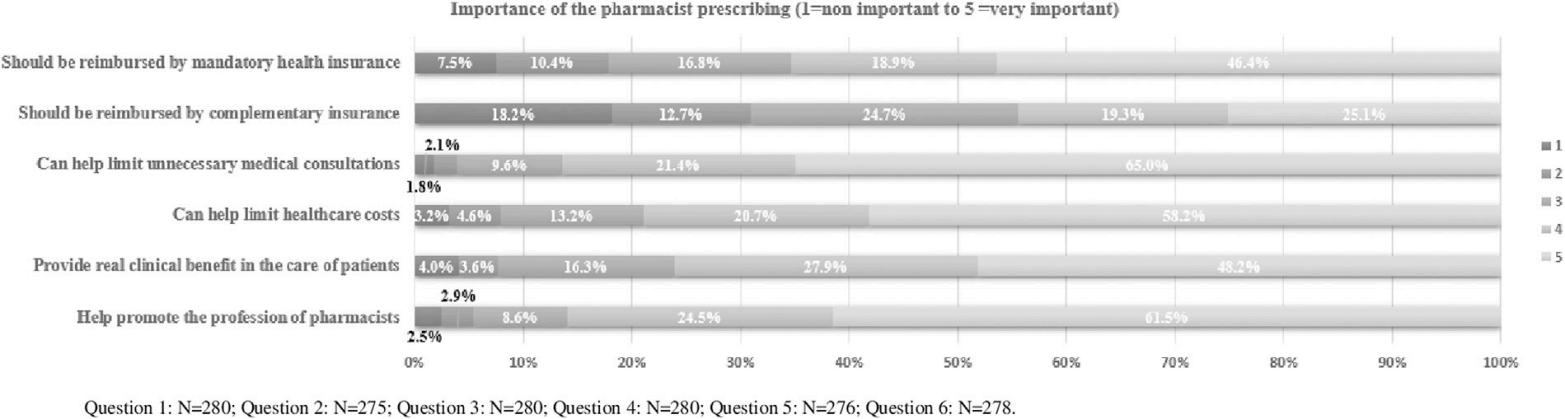
Characteristics and importance of the pharmacist prescribing list perceived by respondent pharmacists.

The opinion of respondent pharmacists about the characteristics and importance of the PP list is included in Figure 3. Most pharmacists (86.4%, n = 242) considered that the PP list could limit unnecessary medical consultations, 78.9% (n = 221) that it could help limit healthcare costs, 76.1% (n = 210) believed it provides a real clinical benefit in patients’ care, and 86% (n = 239) that it could help promoting pharmacists. Most pharmacists consider that the medications included in the list should be compensated either by the mandatory (65.3%, n = 183) or complementary healthcare insurance (44.4%, n = 122).

Most pharmacist did not respond when their opinion on additional medications to be included in the PP list was asked (66.9%, n = 184). Some pharmacists (12.7%, n = 35) thought that no medications should be added. Among the 56 (20.4%) who specified additional medications should be include: antibiotics (17.8%, n = 10), oral contraception (10.7%, n = 6), all the medications under medical prescription (8.9%, n = 5), oral corticosteroids (7.1%, n = 4), antimalarial (3.6%, n = 2), myorelaxants (3.6%, n = 2), antidiabetics (3.6%, n = 2) and vaccines (1.8%, n = 1).

## Discussion

### Objective 1. to evaluate the clinical relevance of the medications included in the PP list compared to OTC medications

To our knowledge, this is the first study to analyze the clinical relevance of a PP list. To increase the contribution of community pharmacists in Primary Care and to broad their scope of practice, it was important to understand the added value of the PP list for treating patients in community pharmacies compared to the medications that were already available. We believe that the method presented in this study could be replicated in other contexts to identify which medicines are clinically relevant to the management of patients through autonomous pharmacists prescribing. After the analysis of the list relevance, only 19.0% (20 medications with 14 different drug indications) of the products included in the list were considered to provide a real benefit to patients’ care compared to medications already available in OTC. Reasons for limited clinical relevance could be that several medications are identical or similar to options already approved and available in pharmacies as OTCs or are part of homogenous therapeutic classes that already include OTC medications, such as proton pump inhibitors for gastro-esophageal reflux (e.g., omeprazole, pantoprazole) or antihistamines for seasonal allergic rhinitis or urticaria (e.g., cetirizine, fexofenadine). This is a low percentage to achieve the desired goal of the medication reclassification, such as limiting medical consultations or health costs. Also, six medications were already available as non-prescription medication but were reclassified for safety reasons as prescription medication that could also be prescribed by pharmacists. In addition, several of the 14 drug indications treated by one of the 20 medications considered first-line treatment are rare (e.g., scabies or emergency treatment of an opioid overdose–to be noted that this latter has been withdraw from the PP list in June 2023) which limits the use of those medications. Furthermore, the active ingredient mebeverine was considered as having a positive clinical relevance because it offers a new option of treatment for pharmacists compared to OTC medications, nevertheless, its efficacy is not well established. Since medications with a negative clinical relevance represented 9.5% of the total, the inclusion of these products in the PP list should be revised.

### Objective 2. to evaluate the implementation of the medications included in the PP list into patient’s daily care in community pharmacy

The second objective of the study was analyzing the use of the medications from the PP list in community pharmacies and the pharmacists’ opinion. The number of respondents in each region could be explained by the total number pharmacies in each area (OFS, 2021). A research from the University of Basel (Giuranno et al., 2021) consisted in a questionnaire for community pharmacists took place on the same year in the German speaking regions on this topic and could explain why a part of responders chose not to answer the questionnaire. Both studies allow to complete the view on the use of PP list, as our study mostly include answers from French and Italian speaking pharmacists that were not included in this previous research from Basel.

Almost half of the pharmacies reported to have prescribed between 10 and 50 medications from the PP list in the last 6 months and 30% of the total respondents prescribed under 10 medications. This is lower, also when a sub analysis of the respondents from the German region was carried out, compared to the results obtained in the study from Basel (Giuranno et al., 2021) where it was reported that 35% of the pharmacies (n = 217) used to deliver medications from PP list several times per week and a further 35% reported to deliver these several times per month. This result illustrates the lower implementation of the medications included in the PP list in patients’ care in the French and Italian speaking part of Switzerland which may be related to the differences in dispensing between the German speaking part (where medical practitioners are allowed to dispense medication in most of the regions) and the French and Italian speaking part (where only community pharmacists can dispense medication). Indeed, in regions where medical practitioners cannot dispense medication, community pharmacists are concerned about the opinions of GPs in relation to the autonomous pharmacists prescribing (Matthey de l’Endroit, 2022).

The most important drug indication according to pharmacists was emergency contraception, which is one of the medications with positive clinical relevance since levonorgestrel is the first choice for treatment. The second most important drug indication was bacterial conjunctivitis (eye disease) treated by hexamidine that also had a positive clinical relevance. These results could be related to patients’ demands, because patients were still familiar to both medications that were non-prescription medication reclassified for safety reasons as prescription medication that could also be prescribed by pharmacists.

Pharmacists believed that certain treatments should be added to the list, most of them named the antibiotics for systemic use. Pharmacist diagnosing and managing acute common infections (e.g., cystitis) could limit the number of medical consultations and ultimately health costs. Such competencies for community pharmacists are now included in countries such as Australia since 2022 (The Guild of Australia, 2022). The second therapeutic class of medications demanded by pharmacists to be included in the PP list was oral hormonal contraception, as found in other studies (Yous et al., 2020; Eckhaus et al., 2021). This is in line with practice observed in other countries such as United States (Grossman and Fuentes, 2013) or Canada (Navarrete et al., 2022) where the service has shown users’ acceptability and reach (Navarrete et al., 2021). Nevertheless, when GPs were asked through a study carried out in Switzerland, concerns about patients’ safety aroused although combined access model (initial prescription from GPs and follow-up prescriptions by pharmacists) found acceptance (Yous et al., 2021).

In Switzerland, community pharmacists are already authorized to deliver treatments such as oral antibiotics or oral hormonal contraception in some specific conditions, for example, if delivery is intended to: avoid a direct danger, relieve acute symptoms that require immediate intervention or allow the continuation of a prescribed treatment that should not be interrupted (Hersberger and Beutler, 2010). The request by pharmacists to add such medications to the list can be interpreted as a way of clarifying their role and responsibilities under these conditions and facilitating a practice that already exists.

In general, when PP has been studied from patients or any other stakeholders, common results have been found such as ease of patient access to healthcare, improved patient outcomes, better use of pharmacists’ skills or reduced physician workload. But also, negative aspects have been highlighted such as the lack of access to patient clinical records or limited pharmacist diagnosis skills (Famiyeh and McCarthy, 2017; Jebara et al., 2018; Yous et al., 2021).

From the pharmacists’ perspective, the PP list should ameliorate patients’ care and pharmacists’ practice and limit unnecessary medical consultations and healthcare costs. Nevertheless, in real practice the service was considered to confront numerous barriers (e.g., service not reimbursed by the mandatory health insurance or not sufficient external support to integrate the PP list in daily practice). In clinical situations where the patient could equally be managed with medications from the PP list or OTC, respondent pharmacists chose OTC on most occasions. The low clinical relevance of the medications in the pharmacist prescribing list could partially explain this situation. In addition, pharmacists are used to deliver OTC medications and the service is, at least partly, financed by the margin on the medication. For PP list, pharmacists need to charge a separate fee that might need to be explained to the patient. Hence, they continue to use OTC medications in patients’ care. Also related to costs, most pharmacists considered that the medications included in the list should be compensated either by the mandatory (65.3%) or complementary healthcare insurance (44.4%). Similarly to the results found in the work carried out by the University of Basel (Giuranno et al., 2021). This could help to set a pricing of this service (nowadays the price for the service is freely determined by each pharmacy and usually it is a flat rate of CHF 20–30) and to legitimate it towards the patients. However, as the service is mainly intended to people in good health who do not have a GP and who generally choose a high yearly co-payment according to the Swiss health insurance system, this would probably have little influence on reimbursement to patients (as patients with high co-payment would have to pay out of pocket for the service).

Regarding to the implementation of the service, 39.8% of respondent would need more help through additional training or algorithms. The same results were obtained in the study from University of Basel on the use of the PP list (Giuranno et al., 2021). Nevertheless, from 2022 those requirements were offered for some medications and minor ailments through pharmaSuisse (Pharmasuisse, 2022).

### Study limitations

It is important to notice that the study may have methodological limitations such as the absence of a systematic review for the evaluation of the clinical relevance of the PP list or few evidence-based data available for some medications treating minor ailments. Nevertheless, the most relevant sources and guidelines in Swiss community pharmacy for the consulted health problems were studied and, except for mebeverine, medications had a well-defined clinical relevance in the different guidelines. As these guidelines refer to international clinical studies or are edited by international medical societies, results are not only limited to the Swiss practice. In a conservative approach, medications in the PP list with similar OTC (same active ingredient but different dosage and/or dose form) were not considered in this study as clinically relevant. However, the difference will often be in the dosage, duration of treatment, and/or minimum age for treatment which could also be considered as new therapeutic options for the community pharmacists.

For the second objective of the study, a higher number of answers were obtained by pharmacists from the French and Italian part of Switzerland. Therefore, the study might not represent the whole population of Swiss pharmacists. However, a previous study carried out in the German regions showed similar results.

## Conclusion

The Swiss PP list seems limited to achieve its goals of reducing medical consultations and healthcare costs. Most first-line treatments available in the PP list are already available as OTCs. However, this illustrates that pharmacists are trusted to correctly assess the clinical relevance even when first-line treatments are not an option. Pharmacists highlight the importance of prescribing medications from this list to achieve this goal; however, its use was not implemented after 3 years.

To better integrate medications from the PP list in patients’ daily care, a revision to enhance its clinical relevance would be recommended. Other barriers found to the PP list implementation such as pharmacists’ training or medications costs for patients could also be considered by policymakers.

## Data Availability

The raw data supporting the conclusion of this article will be made available by the authors, without undue reservation.

## References

[B1] Ahpra (2019). Pharmacist prescribing - position statement - 15 october 2019 ahpra and national boards. Sydney (New South Wales) 2001, Australia: Pharmacy Board Ahpra. Available from: www.pharmacyboard.gov.au/News/Professional-Practice-Issues/Pharmacist-Prescribing-Position-Statement.aspx.

[B2] Aly M. García-Cárdenas V. Williams K. Benrimoj S. I. (2018). A review of international pharmacy-based minor ailment services and proposed service design model. Res. Soc. Adm. Pharm. 14 (11), 989–998. 10.1016/j.sapharm.2017.12.004 29444752

[B3] Amador-Fernández N. B. S. García-Mochón L. García-Cárdenas V. Dineen-Griffin S. Gastelurrutia M. A. (2021). A cost utility analysis alongside a cluster-randomised trial evaluating a minor ailment service compared to usual care in community pharmacy. BMC Health Serv. Res. 21 (1), 1253. 10.1186/s12913-021-07188-4 34798895 PMC8605551

[B4] CDC (2022). Health insurance portability and accountability act of 1996 (HIPAA). Atlanta: Centers for Disease Control and Prevention. Available from: www.cdc.gov/phlp/publications/topic/hipaa.html#:∼:text=The%20Health%20Insurance%20Portability%20and,the%20patient’s%20consent%20or%20knowledge.

[B5] Dineen-Griffin S. Benrimoj S. I. Rogers K. Williams K. A. Garcia-Cardenas V. (2020a). Cluster randomised controlled trial evaluating the clinical and humanistic impact of a pharmacist-led minor ailment service. BMJ Qual. Saf. 29 (11), 921–931. 10.1136/bmjqs-2019-010608 32139400

[B6] Dineen-Griffin S. Vargas C. Williams K. A. Benrimoj S. I. Garcia-Cardenas V. (2020b). Cost utility of a pharmacist-led minor ailment service compared with usual pharmacist care. Cost. Eff. Resour. Alloc. 18, 24. 10.1186/s12962-020-00220-0 32742199 PMC7388462

[B7] Eckhaus L. M. Ti A. J. Curtis K. M. Stewart-Lynch A. L. Whiteman M. K. (2021). Patient and pharmacist perspectives on pharmacist-prescribed contraception: a systematic review. Contraception 103 (2), 66–74. 10.1016/j.contraception.2020.10.012 33130109 PMC11283818

[B8] Famiyeh I. M. McCarthy L. (2017). Pharmacist prescribing: a scoping review about the views and experiences of patients and the public. Res. Soc. Adm. Pharm. 13 (1), 1–16. 10.1016/j.sapharm.2016.01.002 26898951

[B9] Fedlex (2022). Federal act on research involving human beings (human research act, HRA). Bern: Swiss Confederation. Available from: www.fedlex.admin.ch/eli/cc/2013/617/en.

[B10] FGSC (2022). Self-care readiness index 2.0. Nyon: Global Self-Care Federation. Available from: https://selfcarepromise.org/self-care-readiness-index/scri-2022/.

[B11] FOPH (2019). Simplified supply of medicinal products subject to prescription Swiss confederation federal office of public health. Available from: www.bag.admin.ch/bag/en/home/medizin-und-forschung/heilmittel/abgabe-von-arzneimitteln.html (Accessed March 30, 2022).

[B12] Giuranno M. von Wartburg E. Alleman S. (2021). Liste B+: comment est-elle utilisée en pharmacie? B+ list: how is it used in pharmacy?]. Pharma J. 09, 16–18.

[B13] Grossman D. Fuentes L. (2013). Over-the-counter access to oral contraceptives as a reproductive healthcare strategy. Curr. Opin. obstetrics Gynecol. 25 (6), 500–505. 10.1097/GCO.0000000000000019 24121600

[B14] HCI Solutions (2022a). Compendium. CH-3000 Bern, Switzerland: HCI Solutions SA. Available from: https://compendium.ch/(Accessed January 06, 2022).

[B15] HCI Solutions (2022b). Pharmavista. CH-3000 Bern, Switzerland: HCI Solutions SA. Available from: https://pharmavista.ch/(Accessed January 06, 2022).

[B16] Hersberger K. E. Beutler M. (2010). Remise urgente de médicaments sans prescription médicale [Urgent delivery of medication without a medical prescription]. Pharma J. 16, 8–11.

[B17] Jebara T. Cunningham S. MacLure K. Awaisu A. Pallivalapila A. Stewart D. (2018). Stakeholders’ views and experiences of pharmacist prescribing: a systematic review. Br. J. Clin. Pharmacol. 84 (9), 1883–1905. 10.1111/bcp.13624 29873098 PMC6089831

[B18] Jones R. White R. Armstrong D. Ashworth M. Peterset M. (2010). Managing acute illnesses: an enquiry into the quality of general practice in England. London: The King’s Fund.

[B19] Matthey de l’Endroit J. (2022). L’implémentation de la liste B+ dans les pharmacies communautaires. Master thesis. Genève: Université de Genèver.

[B20] Navarrete J. Hughes C. A. Yuksel N. Schindel T. J. Makowsky M. J. Yamamura S. (2022). Community pharmacists’ provision of sexual and reproductive health services: a cross-sectional study in Alberta, Canada. J. Am. Pharm. Assoc. 62, 1214–1223. 10.1016/j.japh.2022.01.018 35153160

[B21] Navarrete J. Yuksel N. Schindel T. J. Hughes C. A. (2021). Sexual and reproductive health services provided by community pharmacists: a scoping review. BMJ open 11 (7), e047034. 10.1136/bmjopen-2020-047034 PMC8314704 34312200

[B22] OFS (2021). Effectif et densité des médecins, des cabinets dentaires et des pharmacies, par canton. CH-2010 Neuchâtel, Switzerland: Office Fédéral de la statistique. Available from: https://www.bfs.admin.ch/bfs/fr/home/statistiques/sante/systeme-sante/autres-prestataires.assetdetail.20044855.html (Accessed June 20, 2022).

[B23] Ogundipe A. Sim T. F. Emmerton L. (2023). Health information communication technology evaluation frameworks for pharmacist prescribing: a systematic scoping review. Res. Soc. Adm. Pharm. 19 (2), 218–234. 10.1016/j.sapharm.2022.09.010 36220754

[B24] Paudyal V. Watson M. C. Sach T. Porteous T. Bond C. M. Wright D. J. (2013). Are pharmacy-based minor ailment schemes a substitute for other service providers? A systematic review. Br. J. General Pract. 63 (612), e472–e481. 10.3399/bjgp13X669194 PMC3693804 23834884

[B25] Pharmasuisse (2021). Faits et chiffres. Pharmacies suisses. Berne: Société Suisse des Pharmaciens. Available from: https://www.pharmasuisse.org/data/docs/fr/45138/Faits-et-chiffres-pharmaSuisse-2021.pdf?v=1.0#:∼:text=Fin%202019%2C%20la%20Suisse%20comptait,affiliation%20de%2083%2C3%20%25.&text=Des%20pharmacies%20ind%C3%A9pendantes%20se%20r%C3%A9unissent,pour%20d%C3%A9gager%20des%20syner%2D%20gies (Accessed February 23, 2022).

[B26] Pharmasuisse (2022). Consultation en pharmacie (Liste B+). CH-3097 Liebefeld, Switzerland: PharmaSuisse. Available from: https://www.pharmasuisse.org/fr/2065/Consultation-en-pharmacie-Liste-B.htm (Accessed June 18, 2022).

[B27] Rafferty E. Yaghoubi M. Taylor J. Farag M. (2017). Costs and savings associated with a pharmacists prescribing for minor ailments program in Saskatchewan. Cost Eff. Resour. Alloc. 15, 3. 10.1186/s12962-017-0066-7 28400708 PMC5387257

[B28] The Guild of Australia (2022). UTI program now permanent in Queensland the pharmacy Guild of Australia the pharmacy Guild of Australia. Available from: https://www.guild.org.au/news-events/news/forefront/v12n10/uti-program-now-permanent-in-qld#:∼:text=%E2%80%9CFrom%201%20October%202022%2C%20women,or%20visiting%20an%20emergency%20department.%E2%80%9D.

[B29] Vanderbilt (2023). REDCap: research electronic capture. Tennessee: Vanderbilt University. Available from: https://projectredcap.org/.

[B30] Watson M. C. Ferguson J. Barton G. R. Maskrey V. Blyth A. Paudyal V. (2015). A cohort study of influences, health outcomes and costs of patients’ health-seeking behaviour for minor ailments from primary and emergency care settings. BMJ Open 5 (2), e006261. 10.1136/bmjopen-2014-006261 PMC4336457 25694456

[B31] Yous T. Allemann S. Lutters M. (2020). Extended access to hormonal contraception in pharmacies: a survey among Swiss pharmacists. Pharm. (Basel, Switz.) 8 (4), 210. 10.3390/pharmacy8040210 PMC7712984 33182547

[B32] Yous T. Allemann S. Lutters M. (2021). Physicians’ opinion regarding extended access to hormonal contraception in Switzerland. Pharm. (Basel, Switz.) 9 (4), 184. 10.3390/pharmacy9040184 PMC8628942 34842813

