# Supplementary material for: Clinical relevance and implementation into daily practice of pharmacist-prescribed medication for the management of minor ailments
Source: Front Pharmacol. 2024 Jan 24;14:1256172. doi: 10.3389/fphar.2023.1256172 (PMC10851422; doi:10.3389/fphar.2023.1256172)
Supplement: Supplementary file 1 [file DataSheet1.PDF]

Appendix 1: Questionnaire

Since January 2019, pharmacists can autonomously prescribe prescription drugs under certain conditions. Pharmacists have a recognized role as first-line health specialists in the management of frequent illnesses.
We are seeking to better understand how pharmacists integrate the use of drugs "that can be dispensed without a prescription for the treatment of frequent diseases" into their practice in pharmacy. In this questionnaire, these drugs will be called "pharmacist prescribing list or PP list" and "consultation by the pharmacist" will be used to talk about the management of patients by deliverying these drugs.
Thank you in advance for your participation. The questionnaire is anonymous and it should take around 15 minutes to be completed. Several pharmacists working in the same pharmacy can complete it. If you work in multiple pharmacies, please answer based on the pharmacy where your occupancy rate is the highest.

1. Canton of the pharmacy where you work?

AG	AI	AR	BE	BL	BS	FR	GE	GL	GR	JU	LU	NE
NW	OW	SG	SH	SO	SZ	TG	TI	UR	VD	VS	ZG	ZH

2. How do you qualify the pharmacy where you work?

Independent

Part of a chain

Part of a group

Under franchise

3. Your pharmacy is located in:

City

Suburb (outskirts of a city)

Commercial area (uninhabited part of a city / town)

Countryside (village)

4. Which source do you consult primarily to identify the PP drug?

Documenta from i.m@il-offizin (reserved for subscribers)

OFSP website

PharmaSuisse data sheets (members only)

Pharmacy computer system

None

Don't know / don't want to answer

Other:

5.How did the pharmacy where you work support the integration of the PP list into your pharmacy practice?

By training staff

By making documents (e.g., delivery algorithms) available to staff

By providing support tools (e.g., adapted pharmacy management computer system) for staff

None

Don't know / don't want to answer

Other:

6. Would you need more support to integrate the PP list into your practice?

No

Yes

(if yes) Please specify:

7. How many drugs from the PP list do you consider to have been provided following a "consultation by the pharmacist" in your pharmacy during the past 6 months (by you and possibly other pharmacists)?

None

1-10

10-50

50-100
> 100
Don't know / don't want to answer

8. Have you ever personally provided a PP list medication following a consultation you have conducted?

Yes

No

(If yes)

What support do you primarily use to guide your consultation ("algorithm")?

ApoHealth

netCare

ProPharmaX

Rockethealth

No

Don't know / don't want to answer

Other:

What support do you primarily use to document your consultation?

Paper support

IT support

No support

Don't know / don't want to answer

Other:

9. In your pharmacy, has a commercial strategy (e.g., commercial objectives / communication campaign) around the PP list been defined?

Yes, at a channel or group level

Yes, at a pharmacy level

No

Don't know / don't want to answer

(if yes)

What are the elements integrated into this commercial strategy?

Objectives in terms of incomes

Goals in terms of number of customers / transactions

Communication with the public (e.g., magazine, website, social networks, posters in the pharmacy, etc.)

Communication with other health professionals

Public information in the pharmacy by presenting the service and the prices applied

Don't know / don't want to answer

10. From all the indications that can currently be supported by the PP list, select the 5 most relevant in your opinion, for your pharmacy practice:

A. Seasonal allergic rhinitis

B. Eye diseases

C. Acute diseases of the respiratory system

D. Diseases of the digestive system

E. Dermatoses

F. Urogenital diseases

G. Difficulty falling asleep

H. Hypotension

I. Travel sickness and dizziness

J. Emergency contraception

K. Migraine crisis

L. Acute pain

M. Smoking cessation

N. Vitamin and mineral deficiencies

O. Caries prophylaxis

P. Emergency treatment of opioid overdose

11. Faced with a clinical situation justifying the delivery of a drug from the PP list, in the presence of a drug available both as OTC and in PP list (e.g., omeprazole or cetirizine), you prefer to recomend a drug:

OTC

What reason (s) explain (s) this choice?

It's easier

It is faster

This does not require to justify the payment of a service

This makes it possible to deliver an equally effective drug

Other :

PP list

What reason (s) explain (s) this choice?

This makes it possible to document / secure the delivery

This enhances the activity of the pharmacist

This brings additional income through the service

This allows a more effective drug to be delivered

Other :

12. When faced a situation of functional disorders of the gastrointestinal tract,

you prefer to perform a standard pharmaceutical triage and issue an OTC drug (e.g., Gaspan® or Iberogast®)

What reason (s) explain (s) this choice?

It's easier

It is faster

This does not require to justify the payment of a service

This makes it possible to deliver an equally effective drug

Other:

a "consultation by the pharmacist" by recommending a drug from the PP list (e.g., Duspatalin®)

What reason (s) explain (s) this choice?

This makes it possible to document / secure the delivery

This enhances the activity of the pharmacist

This brings additional income through the service

This allows a more effective drug to be delivered

Other :

13. Faced with a situation of acute pain, you prefer to perform:

a standard pharmaceutical triage and issue an OTC drug (e.g., Dafalgan® 500mg or Algifor-L®)

What reason (s) explain (s) this choice?

It's easier

It is faster

This does not require to justify the payment of a service

This makes it possible to deliver an equally effective drug

Other:

a "consultation by the pharmacist" by recommending a PP list package (e.g., Novalgin®, Dafalgan 1g®, or other analgesic)

What reason (s) explain (s) this choice?

This makes it possible to document / secure the delivery

This enhances the activity of the pharmacist

This brings additional income through the service

This allows a more effective drug to be delivered

Other :

14.In general, what importance do you give to the following statements about the PP list (0 being not at all important and 5 being very important):

Should be reimbursed by basic health insurance	0	1	2	3	4	5
Should be reimbursed by supplementary insurance	0	1	2	3	4	5
Can help limit unnecessary medical consultations	0	1	2	3	4	5
Can help limit health care costs	0	1	2	3	4	5
Provide real clinical benefit in patients' care	0	1	2	3	4	5
Help promoting the pharmacist profession	0	1	2	3	4	5

15. In your opinion which class (s) of drug (s) should complete the PP list?

None

Don't know / don't want to answer

The following drug (s):
